# Comparison of Incidence or Recurrence of Anterior Uveitis in Patients with Ankylosing Spondylitis Treated with Tumor Necrosis Factor Inhibitors

**DOI:** 10.3390/jcm13030912

**Published:** 2024-02-05

**Authors:** Hyeon Yoon Kwon, Yu Jeong Kim, Tae-Hwan Kim, Seong Joon Ahn

**Affiliations:** 1 Department of Ophthalmology, Hanyang University Hospital, Hanyang University College of Medicine, Seoul 04763, Republic of Korea; kwon822@naver.com (H.Y.K.);; 2 Department of Rheumatology, Hanyang University Hospital for Rheumatic Diseases, Hanyang University College of Medicine, Seoul 04763, Republic of Korea

**Keywords:** anterior uveitis, ankylosing spondylitis, tumor necrosis factor inhibitors

## Abstract

**Background**: Anterior uveitis (AU) is a significant concern in patients with ankylosing spondylitis (AS), and the choice of tumor necrosis factor inhibitors (TNFi) as a treatment modality raises questions regarding its effects on AU. We compared the effects of TNFi on AU in patients with AS. **Methods**: Patients diagnosed with AS and treated with at least one TNFi, including anti-TNFα antibodies (adalimumab and infliximab) or a soluble TNF receptor molecule (etanercept), between January 2010 and December 2022, were retrospectively reviewed. We compared the recurrence rate of AU in patients with a history of uveitis and the incidence of new-onset AU in those without a history of uveitis among the three TNFi groups. We also compared the effects of two different TNFi agents in patients who underwent TNFi switching. **Results**: Within two years of treatment initiation, there was no significant difference in AU recurrence among the three TNFi groups. However, the incidence of new-onset AU was significantly higher in the etanercept group than in the adalimumab group (26.4% vs. 6.3%; *p* = 0.024). After two years, the AU recurrence rate was significantly lower in the adalimumab group than in the other groups (*p* < 0.001). Among patients who underwent anti-TNFi switching, adalimumab treatment was associated with a significantly lower incidence of uveitis than etanercept (*p* = 0.023). **Conclusion**: In the short-term period following TNFi therapy, etanercept induced new-onset AU more frequently than adalimumab in patients with AS. Adalimumab recipients experienced fewer AU recurrences during the subsequent long-term period compared to other TNFi recipients.

## 1. Introduction

Ankylosing spondylitis (AS), classified as a subtype of spondyloarthritis, is a chronic inflammatory disease primarily affecting the spine and sacroiliac joints [1]. Nonsteroidal anti-inflammatory drugs (NSAIDs) are the first-line treatment for AS, providing symptomatic relief and potentially inhibiting disease progression. Disease-modifying anti-rheumatic drugs such as sulfasalazine have been used as secondary therapies, which showed minor benefits in specific situations [2]. Tumor necrosis factor-α inhibitors (TNFi), such as etanercept, infliximab, and adalimumab, have gained wide acceptance for alleviating AS-related signs and symptoms and preventing disease recurrence [3].

TNFi block the activity of TNFα, a key inflammatory mediator as a pro-inflammatory cytokine. Several studies have established a significant relationship between TNFα and AS [4,5,6,7,8]. TNFα is known to play a crucial role in the pathogenesis of AS, as it is over-produced in AS predominantly by active macrophages and T-lymphocytes, playing key roles in the inflammation of spine and sacroiliac joints and extra-articular manifestations including uveitis [4,5]. Genetic association studies have evaluated the link between TNFα polymorphisms and susceptibility to AS, indicating a potential genetic influence on the development of the disease [6]. Furthermore, the efficacy of TNFα inhibitors for the treatment of AS has been demonstrated, highlighting the therapeutic relevance of targeting TNFα in managing AS [7,8]. Additionally, TNFα has been identified as a risk factor for AS recurrence, further underlining its clinical significance and prognostic value in the context of AS. By inhibiting TNFα, TNFi modulate the immune system by interfering with TNFα signaling pathways, leading to reduced inflammatory cascade that contributes to joint and extra-articular inflammation in AS and symptom relief. Its effectiveness in AS is often measured by improvements in symptoms, function, and disease activity, for which validated assessment tools, such as the Bath Ankylosing Spondylitis Disease Activity Index (BASDAI) and Bath Ankylosing Spondylitis Functional Index (BASFI) can be used. 

Anterior uveitis (AU) is one of the most prevalent extra-articular manifestations in AS patients, with a 20–30% chance of developing the condition over the course of the disease [9,10], and its prevalence increases as AS progresses. Various guidelines have been established for the use of TNFi in AS-associated AU [11,12,13]; the American Academy of Ophthalmology (AAO) recommends TNF inhibitors as second-line immunomodulatory agents for severe uveitis associated with spondyloarthritis. Additionally, the American College of Rheumatology (ACR) recommends anti-TNFα monoclonal antibodies (adalimumab and infliximab) over soluble TNF receptors (etanercept) to reduce uveitis recurrence [11], and Guidelines from the European League Against Rheumatism (EULAR) state that infliximab and adalimumab effectively prevent uveitis recurrence, while etanercept is considered less effective [12]. 

Several studies have examined the impact of TNFi on AU in patients with AS [7,8,14,15]. For AS-associated AU, the efficacy of TNFi for reducing intraocular inflammation or prevention of uveitis recurrence has been evaluated, and previous studies observed that the majority of uveitis occurrences tend to manifest within the short-term period, specifically the first 2 years following the initiation of TNF inhibitor therapy [7,8]. However, the implications of long-term TNF inhibitor therapy on the risk of uveitis after the initial short-term period remain uncertain. Therefore, this study aims to investigate the recurrence and occurrence of AU in AS patients treated with TNFi during both the short-term and subsequent periods, comparing outcomes among three distinct TNFi agents. Additionally, through additional analyses on a cohort of patients who underwent drug switching, a population underexplored in prior studies, this study intends to offer an insight on the efficacy of different TNFi agents, guiding drug-switching decisions in patients already on TNF inhibitors.

## 2. Materials and Methods 

### 2.1. Patient Selection and Data Collection

We retrospectively reviewed the medical records of 216 patients diagnosed with AS who initiated treatment with etanercept, adalimumab, or infliximab and underwent ophthalmic examinations at Hanyang University Hospital between January 2010 and December 2022. The diagnosis of AS was made by rheumatologists based on clinical features such as chronic (≥3 months) inflammatory back pain, imaging studies (sacroiliitis on X-rays or magnetic resonance imaging), and laboratory tests (HLA-B27 positivity or elevated C-reactive protein), in accordance with the Assessment of SpondyloArthritis International Society (ASAS) classification criteria [16,17]. Exclusion criteria encompassed patients lacking sacroiliac joint imaging, those with a follow-up duration of less than 3 months, and individuals with uveitis attributed to infectious (e.g., viral or bacterial) etiologies. Patients with other underlying conditions such as Behcet’s disease, Vogt–Koyanagi–Harada syndrome, or sarcoidosis were also excluded. Additionally, individuals who discontinued TNFi treatment due to adverse events, including hypersensitivity reactions, were excluded. After excluding 7 patients, the final cohort included 209 patients (*n* = 99, 68, and 42 in the etanercept, adalimumab, and infliximab groups, respectively). We ensured that the sample sizes were sufficient to achieve a specified level of statistical power (α [type I error] = 0.05, power = 0.8). Based on an observed difference (20.9%) in a pilot study involving a limited sample set (*n* = 36), at least 74 samples allocated to the etanercept group and 37 to the adalimumab or infliximab group were required. Collected clinical information included sex, age, type and duration of TNFi treatment, HLA-B27 positivity, presence of AU during follow-up period, detailed uveitis manifestations [18], and timing of new-onset uveitis or recurrence.

Baseline and follow-up examinations included comprehensive ophthalmic assessments such as visual acuity, non-contact tonometry, autorefraction, and slit-lamp biomicroscopy. A comprehensive medical history was obtained from all patients during their initial visit to assess the history and treatment of uveitis. Diagnosis of AU was based on clinical examination by ophthalmologists, with slit-lamp biomicroscopy performed to evaluate the degree of inflammation, including the flares and cells in the anterior chamber. The degree of inflammation in the anterior chamber and vitreous was evaluated according to the criteria set by the Standardization of Uveitis Nomenclature Working Group (SUN) [19]. This study was approved by the Institutional Review Board of the Hanyang University Hospital. The IRB waived the requirement for obtaining informed consent from patients for the retrospective nature of the study. Our study adhered to the ethical principles outlined in the Declaration of Helsinki.

### 2.2. Treatment

Management of acute AU involved the administration of topical steroids (prednisolone acetate 1% ophthalmic solution; Daewoo Pharma. Co., Ltd., Busan, Republic of Korea), and cycloplegics. In severe AU cases with 3+ or higher inflammation, systemic steroid treatment was initiated at 0.5 mg/kg/day, followed by a gradual tapering regimen guided by clinical progress. Subtenon triamcinolone acetonide injections were administered concurrently in cases of uncontrolled or severe intraocular inflammation. The selection and dosage of TNFi for AS treatment were determined by rheumatologists based on individual clinical conditions. Etanercept was administered subcutaneously at a weekly dose of 25 mg or increased to 50 mg twice a week. Adalimumab was administered subcutaneously every 2–6 weeks at a dose of 40 mg, while infliximab was administered intravenously at weeks 0, 2, and 6 at a dose of 5 mg/kg body weight, followed by intervals of 6 weeks.

### 2.3. Analyses

We compared the effects of the three TNFi treatments using several parameters and analyses. First, we calculated the AU rates (percentage of patients experiencing uveitis) for each TNFi during the initial two-year period. Patients were categorized based on their uveitis history, enabling us to differentiate between the “occurrence” of new-onset AU (in those without any history) and the “recurrence” of AU (in those with anterior uveitis history) following treatment initiation. As the follow-up period, particularly after the 2 years of treatment, varied among the patients, we calculated the AU rates per 100 patient-years in the subsequent phase (beyond 2 years of treatment) for each TNFi. Occurrences of new-onset AU and AU recurrence were separately compared for each period among the three TNFi groups.

Additionally, we defined a subgroup called the switching group, comprising patients who were switched from one TNFi to another for therapeutic purposes under the guidance of prescribing physicians (rheumatologists). In this subgroup, we assessed the incidence and recurrence rates of AU between the two TNFi agents for each patient.

All statistical analyses were performed using the Statistical Package for the Social Sciences (SPSS), version 29.0 (IBM Corp., Armonk, NY, USA). Continuous variables are presented as mean ± standard deviation, while categorical variables are reported as numbers (percentages). The proportions between the two groups were compared using the chi-square test or Fisher’s exact test. Analysis of variance (ANOVA) and Welch’s ANOVA were used to compare the differences in the means of continuous variables among the three TNFi groups, considering the equality of variance. Post hoc analysis was conducted using Tukey’s HSD test. Survival analysis using Kaplan–Meier curves was performed to examine the rate of AU recurrence within the first two years and beyond two years. Multivariable logistic analysis was performed to evaluate the factors associated with uveitis occurrence using the demographic and clinical characteristics such as age, TNF inhibitor used, history of anterior uveitis before the TNF inhibitor therapy, and follow-up period. The Wilcoxon signed-rank test was used in the subgroup analysis to compare the two TNFi agents used within each patient in the switching group. Statistical significance was set at *p* < 0.05.

## 3. Results

### 3.1. Demographic and Clinical Characteristics

The demographic and clinical characteristics of the 209 patients included in this study are presented in Table 1. The mean age was 44.6 ± 11.6 years, and 76.6% (*n* = 160) of the patients were men. A total of 197 patients (94.3%) were positive for HLA-B27 antigen. 

Before TNFi treatment, 105 patients (50.2%) had a history of AU. Etanercept, adalimumab, and infliximab were initially used in 47.4% (*n* = 99), 32.5% (*n* = 68), and 20.1% (*n* = 42) of patients, respectively. A total of 26 patients (12.4%) underwent switching of TNFi agents. The median follow-up period was 89.5 months with the interquartile range (IQR) of 47.5–135.0 months. There were no significant differences between the TNFi groups in terms of sex, age, HLA-B27 positivity, history of AU before TNFi initiation, other extra-articular manifestations (i.e., psoriasis and inflammatory bowel disease), or number of recurrences prior to TNFα inhibitor use (Table 2), indicating baseline comparability. Furthermore, there were no significant differences in bilateral/unilateral involvement and additional details on anterior uveitis such as hypopyon and fibrinous exudates among the three groups (all *p* > 0.05).

### 3.2. Rates of Uveitis Recurrence and Incidence of New-Onset Uveitis in Patients with Ankylosing Spondylitis Receiving TNFα Inhibitors

**Figure 1:**
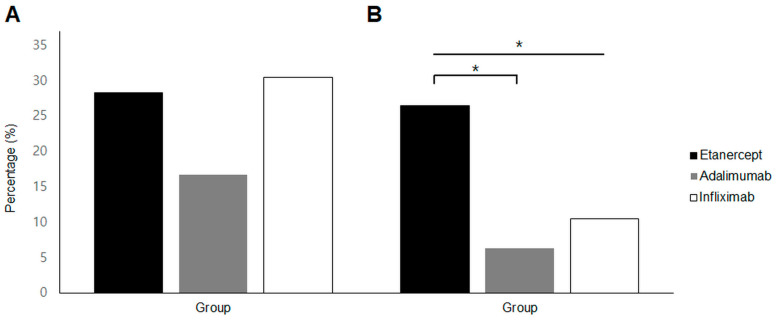
Incidence of uveitis in TNFα inhibitor-users with ankylosing spondylitis in the short-term (within first 2 years of TNFα inhibitor use) phase. (**A**) Recurrence rate in patients with a history of previous uveitis and (**B**) occurrence of new-onset anterior uveitis. * *p* value < 0.05 by Fisher’s exact test.

Among patients with a history of prior uveitis, the percentages of uveitis recurrence during the two years after initiating TNFi therapy were 28.3% (13 patients) for etanercept, 16.7% (6 patients) for adalimumab, and 30.4% (7 patients) for infliximab. No significant differences were observed in the rates of recurrent uveitis among the three groups (*p* = 0.397; Figure 1A).

Regarding new-onset uveitis, during the first two years of TNFi therapy, the percentages of patients developing AU were 26.4% (14 patients) for etanercept, 6.3% (2 patients) for adalimumab, and 10.5% (2 patients) for infliximab. Significant differences were found among the three groups (*p* = 0.040), with the etanercept group showing a higher incidence of new-onset AU compared to the adalimumab group (*p* = 0.024; Figure 1B).

The rates of recurrent uveitis per 100 patient-years during the two-year period of TNFi therapy and the subsequent period were 14.8 and 16.6, respectively (Table 3). There were no significant differences in recurrence rates between these two periods. However, significant differences were observed in the recurrence rates after 2 years among the three TNFi agents (*p* < 0.001). Post hoc analysis revealed significant differences in recurrence rates between etanercept and adalimumab (*p* = 0.014) and between adalimumab and infliximab (*p* = 0.012). 

However, the mean incidence of new-onset uveitis did not show any significant differences among the TNFi groups during or after the first two years, as indicated in Table 4 (both *p* > 0.05). 

### 3.3. Survival and Multivariate Analysis for Occurrence of Uveitis

**Figure 2:**
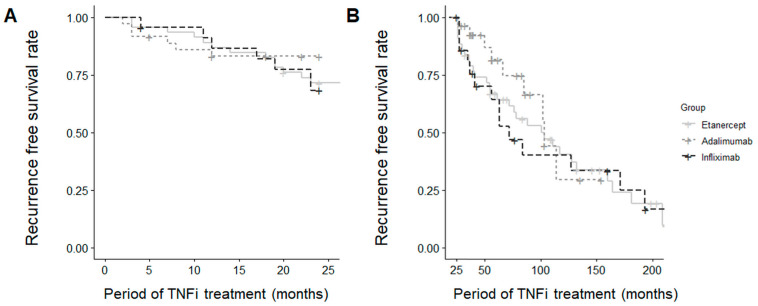
Survival curve for uveitis recurrence (**A**) within the first 2 years and (**B**) beyond 2 years.

Survival analyses showed no significant differences in AU recurrence during the first two years of TNF inhibitor therapy (*p* = 0.549) or the subsequent period (*p* = 0.614) among the three treatment groups (Figure 2). 

**Figure 3:**
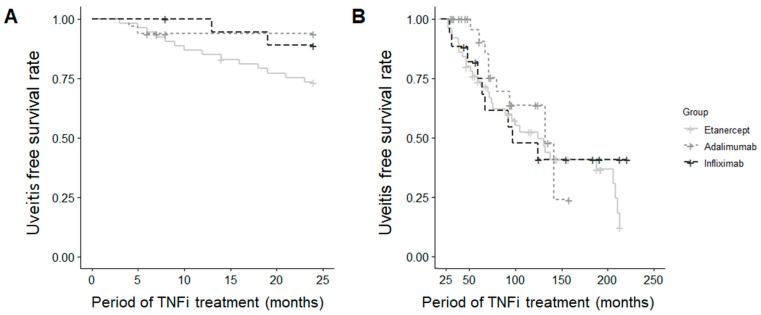
Survival curve for uveitis occurrence (**A**) within the first 2 years and (**B**) beyond 2 years in new-onset cases.

Among patients without a history of uveitis, the incidence of new-onset AU during the first two years (*p* = 0.059) and the subsequent period (*p* = 0.676) of TNFi treatment did not differ significantly among the three groups (Figure 3).

Table 5 presents a multivariate logistic analysis, showing that a history of AU before TNFi therapy significantly increases the risk (Odds Ratio [OR] = 2.00), and the TNFi agents used (adalimumab vs. etanercept) also impacts the occurrence of anterior uveitis. Additionally, a longer follow-up period slightly increases the risk with the OR of 1.013.

### 3.4. Comparison of Uveitis Recurrence or Occurrence in the Switching Group

**Figure 4:**
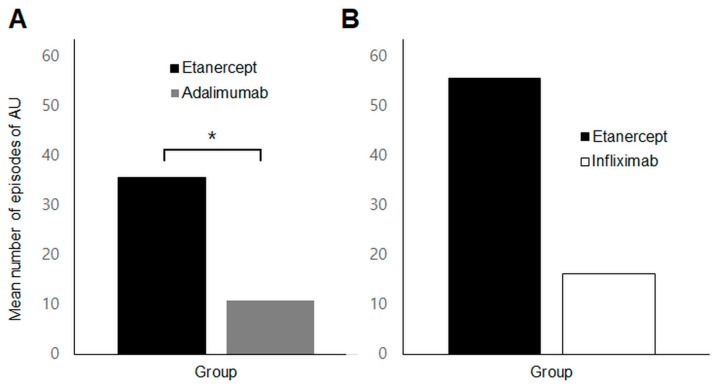
Comparison of the mean number of episodes of anterior uveitis per 100 patient-years in the switching group (**A**) Comparison of etanercept and adalimumab in patients who switched between the two agents (**B**) Comparison of etanercept and infliximab in those who switched between the two. * *p* value < 0.05.

In the subgroup analysis of patients who switched TNFi agents (*n* = 26), a significantly higher incidence of AU was observed during etanercept treatment compared to adalimumab (35.6 ± 38.8 vs. 10.9 ± 19.7 per 100 patient-years, *p* = 0.023) (Figure 4A). However, there was no significant difference in uveitis occurrence/recurrence between etanercept and infliximab treatment periods (*p* = 0.686) (Figure 4B).

## 4. Discussion

This study investigated the rates of uveitis recurrence and new-onset occurrence among patients with AS receiving TNFα inhibitors and examined whether there were differences among three different TNFi agents. Understanding the impact of TNFi treatment on uveitis is crucial in patients with AS, as uveitis, a common extra-articular manifestation of AS, can significantly affect patients’ quality of life [20,21,22], and TNFi treatment is widely used for AS. Our study showed that, during the first two years of therapy, the incidence of new-onset AU was significantly higher in patients treated with etanercept than in those treated with adalimumab. 

Previous reports have provided conflicting findings regarding the efficacy of etanercept in preventing AU incidents [7,23]. While some studies have suggested its effectiveness, others have reported a paradoxical effect with an increased incidence of AU [3,14,24,25,26]. Regarding comparative efficacy with other TNFi agents, a comprehensive investigation using data from the Swedish biologics register revealed a significantly higher risk of anterior uveitis associated with etanercept compared to adalimumab or infliximab in AS patients [14]. Similar findings were reported in other Asian studies, emphasizing the inferiority of etanercept in preventing uveitis [7,8]. Recognizing these discrepancies, certain guidelines acknowledge the potential advantages of monoclonal antibodies, such as adalimumab and infliximab over soluble TNF receptors, and recommend the antibodies for the treatment of AS due to concerns regarding AU [11,12,13]. 

For detailed comparative analyses, our study separately analyzed the rates of new-onset uveitis or recurrence in two distinct periods: the initial two years following the initiation of TNFi therapy and the subsequent period. This study aimed to differentiate between the short- and long-term outcomes of new-onset uveitis and uveitis recurrence following TNFi therapy, as previous studies have predominantly focused on short-term new-onset uveitis following etanercept treatment, thus leaving the long-term impact of etanercept on uveitis recurrence and new-onset uveitis unclear. Our findings regarding uveitis recurrence rates revealed no significant differences among the three treatment groups within the 2-year period following TNFi initiation, suggesting similar short-term outcomes for all three TNFi agents. However, a significant difference between the groups emerged in the subsequent period, indicating that adalimumab may help effectively mitigate the long-term recurrence of uveitis in patients with AS. Multivariable analyses showed no significant association in uveitis recurrence between adalimumab and infliximab but significant association between adalimumab and etanercept. The etanercept group exhibited a higher incidence of new-onset AU compared to the adalimumab group during the first 2 years of TNFi therapy. These findings align with those of previous studies demonstrating more frequent occurrences of new-onset uveitis after etanercept treatment and decreased long-term recurrence rates with anti-TNFα monoclonal antibodies [25,26,27,28].

To validate the findings derived from the comparison of the three TNFi, we conducted additional analyses on patients who underwent drug switching. Among patients who received both etanercept and adalimumab, a significantly lower incidence of AU was observed during adalimumab use (*p* = 0.023). By contrast, no significant difference was observed with other drugs. This analysis enabled us to compare the effects of changing medications in each patient, providing stronger evidence regarding the occurrence of uveitis between the two TNFi agents. The results obtained from these switching cases further corroborated the findings of the comparative analyses of the three treatment groups. Both analyses resulted in consistent findings with previous research highlighting the efficacy of anti-TNFα monoclonal antibodies for the treatment or prevention of uveitis [29,30,31]. Furthermore, our findings from switched cases may further suggest that etanercept can be switched to monoclonal antibodies such as adalimumab, should recurrent anterior uveitis be better controlled. This was compatible with previous finding that patients with a uveitis flare before drug switching did not recur afterwards [8]. 

Thus, our study’s findings have important clinical implications regarding the use of TNFi for the treatment of AU associated with AS. Specifically, our results suggest that adalimumab may be particularly beneficial for AS patients with recurrent AU who require a sustained control of uveitis and prevention of recurrences. On the other hand, AS patients without a history of uveitis receiving etanercept may require more careful monitoring for anterior uveitis compared to those receiving adalimumab, especially during the short-term period, for the higher risk of new-onset uveitis. Additionally, the varying efficacies of different TNFi agents in reducing uveitis should be considered when clinicians consider switching their therapy for patients with recurrent uveitis to different TNFi agents.

This study had some limitations. First, the study design was retrospective, which may have introduced inherent biases and limitations in data collection. In addition, there is a possibility that the number of recurrent AU episodes might have been under-reported, as patients might have visited other clinics for the treatment of recurrent uveitis, although we thoroughly reviewed such histories or episodes from medical records. Additionally, the study may be subject to selection bias inherent in retrospective studies because of the non-randomized nature of the choice of three TNFi agents and the exclusion of patients who were lost to follow-up examinations. Furthermore, other anti-TNF agents, aside from the three agents under our investigation, demonstrated efficacy in reducing the occurrence of uveitis in patients with ankylosing spondylitis. For example, the occurrence rate of acute anterior uveitis significantly decreased from 11.1 before golimumab therapy to 2.2 after the therapy per 100 patient-years [32], and the rate of uveitis flare was lower for those treated with certolizumab than those with placebo [33]. Unfortunately, we were unable to assess the impact of other agents due to the unavailability of relevant data. Moreover, our study has multiple comparisons; therefore, the family-wise error rate (FWER), the probability of making one or more false discoveries, should be considered. As our study had a small number of multiple comparisons (at most 9 for one analysis), for which corrections for multiple comparisons such as Bonferroni or Tukey method had not been applied, our results should be carefully interpreted with a type I error (false positive rate). Ideally, a sample size calculation at the time of study design is required to ensure sufficient statistical power to detect differences between treatments. Future studies should incorporate robust sample size calculations during the study design phase to enhance the reliability and interpretability of the results. Finally, this study did not investigate other potential factors or important biomarkers, such as the disease activity of AS and treatment adherence, which might have influenced the rates of uveitis recurrence and occurrence [5].

## 5. Conclusions

While acknowledging the limitations of this study, this study offers insights into uveitis recurrence and the incidence of new-onset uveitis in patients with AS undergoing TNFi therapy. Our findings suggest a lower risk of uveitis recurrence associated with anti-TNFα monoclonal antibodies, as opposed to an increased risk of recurrence or occurrence with etanercept, both in the short- and long-term periods. These observations hold significant implications for treatment decision making, particularly in choosing TNFi agents for AS treatment, especially in individuals with a history of uveitis episodes. Our findings in the drug-switching population also carry practical implications, suggesting a consideration for transitioning from etanercept to anti-TNFα monoclonal antibodies as a potential treatment strategy for recurrent anterior uveitis in patients with AS. Nevertheless, to confirm and broaden these results, further prospective studies with larger sample sizes are warranted. Such studies can also explore alternative TNFi agents or additional therapeutic options that may contribute to enhancing patient outcomes.

## Figures and Tables

**Table 1:** Demographic and clinical characteristics of patients with ankylosing spondylitis (AS) treated with tumor necrosis factor-α (TNFα) inhibitors.

Characteristics	Total (*n* = 209)
Age, year	44.6 ± 11.6
Sex, male	160 (76.6%)
HLA-B27 positivity	197 (94.3%)
**Extra-articular manifestations**	
History of anterior uveitis before TNFα inhibitor therapy	105 (50.2%)
Psoriasis	30 (14.4%)
Inflammatory bowel disease	8 (3.8%)
**Initial TNFα inhibitor used for treatment**	
Etanercept	99 (47.4%)
Adalimumab	68 (32.5%)
Infliximab	42 (20.1%)
Follow-up period following TNFα inhibitor therapy, median (Q1–Q3) months	89.5 (47.5–135.0)
**Uveitis manifestations of previous episodes**	(% among 105 uveitic patients)
Unilateral/bilateral involvement	65:40 (61.9%:38.1%)
Hypopyon	4 (3.8%)
Fibrinous exudates	8 (7.6%)
Synechiae	24 (22.9%)

**Table 2:** Demographic and clinical characteristics of ankylosing spondylitis patients, categorized by tumor necrosis factor-α (TNFα) inhibitors used at baseline.

Characteristics	Etanercept (*n* = 99)	Adalimumab (*n* = 68)	Infliximab (*n* = 42)	*p*-Value
Sex, male	77 (77.8%)	50 (73.5%)	33 (78.6%)	0.769 ^a^
Age, year (range)	45.2 ± 11.7 (18.0–80.0)	43.2 ± 12.0 (23.0–75.0)	45.2 ± 11.0 (28.0–71.0)	0.319 ^b^
HLA-B27 positivity	95 (96.0%)	63 (92.6%)	39 (92.9%)	0.583 ^c^
Anterior chamber inflammation grade at active uveitis	1.98 ± 1.08	1.67 ± 1.27	2.50 ± 1.39	0.292 ^b^
Number of previous episodes of anterior uveitis prior to the use of TNFα inhibitor	0.65 ± 1.42 (0–10)	1.05 ± 1.41 (0–6)	0.84 ± 1.90 (0–10)	0.079 ^b^
Frequency of previous episodes of anterior uveitis per year	0.91 ± 0.82	1.03 ± 0.88	0.82 ± 0.90	0.624 ^b^
**Extra-articular manifestations**				
Anterior uveitis prior to TNFα inhibitor therapy	46 (46.5%)	36 (52.9%)	23 (54.8%)	0.575 ^a^
Psoriasis	18 (18.2%)	8 (11.8%)	4 (9.5%)	0.378 ^c^
Inflammatory bowel disease	4 (4.0%)	2 (2.9%)	2 (4.8%)	0.900 ^c^
**Uveitis manifestations (*n* = 105)**				
Unilateral/bilateral	26:20 (56.5%:43.5%)	25:11 (69.4%:30.6%)	14:9 (60.9%:39.1%)	0.487 ^a^
Hypopyon	2 (4.3%)	1 (2.8%)	1 (4.3%)	0.999 ^c^
Fibrinous exudates	4 (8.7%)	2 (5.6%)	2 (8.7%)	0.806 ^c^
Synechiae	12 (26.1%)	7 (19.4%)	5 (21.7%)	0.767 ^a^

Data are expressed as Mean ± SD (Min–Max) in continuous variables and *n* (%) in categorical variables. ^a^ Chi-square test. ^b^ Kruskal–Wallis test. ^c^ Fisher’s exact test.

**Table 3:** Comparison of the mean number of recurrences of uveitis per 100 patient-years in the short-term period (within 2 years) and subsequent long-term period (after 2 years).

TNFα Inhibitor	Mean Number of Recurrences within 2 Years (per 100 Patient-Years)	Mean Number of Recurrences after 2 Years (per 100 Patient-Years)	*p*-Value
Overall	14.8	16.6	
Etanercept	18.5	21.1	0.702
Adalimumab	9.7	6.1	0.420
Infliximab	15.2	24.5	0.327
*p* value among 3 TNFi groups	0.483 *	<0.001 ^†^	

* *p* value between the three groups using analysis of variance. ^†^ *p* value between the three groups, using Welch’s analysis of variance.

**Table 4:** Comparison of the mean number of incidences of new-onset uveitis within 2 years and after 2 years (per 100 patient-years).

TNFα Inhibitor	Mean Number of Incidences within 2 Years (per 100 Patient-Years)	Mean Number of Incidences after 2 Years (per 100 Patient-Years)	*p*-Value
Overall	12.5	11.7	
Etanercept	17.9	14.2	0.201
Adalimumab	4.7	5.9	0.353
Infliximab	10.5	14.7	0.301
*p* value among 3 TNFi groups *	0.125	0.118	

* Using analysis of variance.

**Table 5:** Multivariate logistic analysis for identifying the clinical factors associated with the occurrence of anterior uveitis.

Variables	Odds Ratio (95% CI)	*p*-Value
Age	0.974 (0.947–1.001)	0.053
Initial TNF inhibitor used for treatment		**0.049**
Adalimumab vs. Etanercept	0.404 (0.194–0.841)	**0.015**
Infliximab vs. Etanercept	0.797 (0.354–1.794)	0.583
History of anterior uveitis	2.004 (1.050–3.824)	**0.035**
Follow-up period	1.013 (1.005–1.020)	**0.001**

CI, confidence interval; TNF, tumor necrosis factor.

## Data Availability

The datasets generated or analyzed during this current study are available from the corresponding author on reasonable request.
